# Mass Spectrometry Detection of G3m and IGHG3 Alleles and Follow-Up of Differential Mother and Neonate IgG3

**DOI:** 10.1371/journal.pone.0046097

**Published:** 2012-09-25

**Authors:** Célia Dechavanne, François Guillonneau, Giovanni Chiappetta, Laïla Sago, Prisca Lévy, Virginie Salnot, Evelyne Guitard, François Ehrenmann, Cédric Broussard, Philippe Chafey, Agnès Le Port, Joëlle Vinh, Patrick Mayeux, Jean-Michel Dugoujon, Marie-Paule Lefranc, Florence Migot-Nabias

**Affiliations:** 1 Unité Mixte de Recherche (UMR) 216 Mère et enfant face aux infections tropicales, Institut de Recherche pour le Développement (IRD), Paris, France; 2 Faculté de Pharmacie, Université Paris Descartes, Sorbonne Paris Cité, Paris, France; 3 Plate-forme protéomique de l’Université Paris Descartes, Sorbonne Paris Cité, Paris, France; 4 Unité de Spectrométrie de Masse Biologique et Protéomique (SMBP), Unité de Service et de Recherche (USR) 3149 CNRS, Ecole Supérieure de Physique et de Chimie Industrielles (ESPCI) ParisTech, Paris, France; 5 Laboratoire d'Anthropologie Moléculaire et Imagerie de Synthèse, Unité Mixte de Recherche (UMR) 5288, CNRS, Université Paul Sabatier Toulouse III, Toulouse, France; 6 The international ImMunoGeneTics information system® (IMGT®) Laboratoire d’ImmunoGénétique Moléculaire (LIGM), Université Montpellier 2, Unité Propre de Recherche (UPR) CNRS 1142, Institut de Génétique Humaine (IGH), Montpellier, France; 7 Plate-forme électrophorèse Bidimensionnelle Institut Cochin (PeBIC), Inserm Unité 1016, Paris, France; 8 Inserm Unité 1016, Département d’Hématologie, Institut Cochin, Université Paris Descartes, Sorbonne Paris Cité, CNRS, Paris, France; 9 Institut Universitaire de France, Paris, France; Emory University School of Medicine, United States of America

## Abstract

Mass spectrometry (MS) analysis for detection of immunoglobulins (IG) of the human IgG3 subclass is described that relies on polymorphic amino acids of the heavy gamma3 chains. IgG3 is the most polymorphic human IgG subclass with thirteen G3m allotypes located on the constant CH2 and CH3 domains of the gamma3 chain, the combination of which leads to six major G3m alleles. Amino acid changes resulting of extensive sequencing previously led to the definition of 19 IGHG3 alleles that have been correlated to the G3m alleles. As a proof of concept, MS proteotypic peptides were defined which encompass discriminatory amino acids for the identification of the G3m and IGHG3 alleles. Plasma samples originating from ten individuals either homozygous or heterozygous for different G3m alleles, and including one mother and her baby (drawn sequentially from birth to 9 months of age), were analyzed. Total IgG3 were purified using affinity chromatography and then digested by a combination of AspN and trypsin proteases, and peptides of interest were detected by mass spectrometry. The sensitivity of the method was assessed by mixing variable amounts of two plasma samples bearing distinct G3m allotypes. A label-free approach using the high-performance liquid chromatography (HPLC) retention time of peptides and their MS mass analyzer peak intensity gave semi-quantitative information. Quantification was realized by selected reaction monitoring (SRM) using synthetic peptides as internal standards. The possibility offered by this new methodology to detect and quantify neo-synthesized IgG in newborns will improve knowledge on the first acquisition of antibodies in infants and constitutes a promising diagnostic tool for vertically-transmitted diseases.

## Introduction

Systemic transfer of maternal antibodies occurs *in utero* across the placenta. It is limited to the immunoglobulins (IG) of the IgG class which are transported across the syncytiotrophoblasts via a specific pathway involving the placental Fc receptor (FCGRT) [1]. This active transport mechanism of maternal IgG to the fetus usually results in about 90% of the maternal serum level of IgG in the full-term newborn at delivery [2]. Plasma IgG concentrations are in the 7–15 g/L range and IgG account for 75% of serum IG in a human adult. They are constituted of IgG1 (60–70%), IgG2 (20–30%), IgG3 (5–8%) and IgG4 (1–3%) [3]. The length of time during which maternal antibodies persist in the infant’s blood depends on the starting antibody concentration at birth. In general, maternal antibodies fall to minimal levels by 4 months of age and the infant’s antibody titres begin to rise from about 6 months of age, following active immunization. Presence in the infant’s plasma of both maternal and intrinsic antibodies hampers the neonatal serological diagnosis. If a direct diagnosis is impossible or is insufficient, the detection of the neonate’s own antibodies can bring essential information especially for vertically-transmitted diseases for which the methods of antigen detection are not reliable. It is the case of parasitic diseases such as toxoplasmosis (causal agent: *Toxoplasma gondii*) and the American trypanosomiasis also named Chagas disease (causal agent: *Trypanosoma cruzi*), for which the determination of specific antibodies neo-synthesized by the newborn would be helpful during the first months of life to diagnose a congenital infection [4]–[6]. The decision to establish or to shorten medical treatments would be facilitated by the observation of specific antibodies elaborated by the newborn. This requires being able to distinguish IG synthesized by the infant from those transferred from the mother.

On the constant regions of their heavy gamma1, gamma2 and gamma3 chains, the IgG1, IgG2 and IgG3 subclasses carry antigenic determinants or Gm (for *gamma marker*) allotypes [7], [8]. Twenty Gm allotypes are currently identified (18 ‘classical’ ones and two ‘surnumerary’ ones). The 18 ‘classical’ Gm allotypes comprise four G1m, G1m (1, 2, 3, 17), one G2m, G2m (23), and thirteen G3m, G3m (5, 6, 10, 11, 13, 14, 15, 16, 21, 24, 26, 27, 28) [8]. The two ‘surnumerary’ allotypes, G1m27 and G1m28, correspond to allotypes demonstrated to be located in gamma1 chains in African populations, instead of being on gamma3, as expected [8], [9]. Except for G1m3 and G1m17 located on the CH1 domain of gamma1, all other Gm allotypes are located on the Fc (on CH2 or on CH3) [8]. The correlation between Gm allotypes and amino acid changes has been possible following the sequencing of gamma chains and/or IGHG genes [10], and the complete nucleotide sequencing of many IGHG3 alleles from individuals homozygous for well characterized G3m alleles [8], [10]–[12]. The thirteen G3m allotypes are inherited in different combinations or G3m alleles (encoded by one or several IGHG3 alleles) [8]. The six prevalent alleles are G3m5* (G3m5,10,11,13,14,26,27), G3m6* (G3m5,6,10,11,14,26,27), G3m24* (G3m5,6,11,24,26), G3m15* (G3m10,11,13,15,27), G3m16* (G3m10,11,13,15,16,27) and G3m21* (G3m21,26,27,28) [8]. Several G3m allotypes depend on the combination of two or even three amino acids, distant on the linear sequence but close to one another in three-dimensional structures (illustrated in the ‘IMGT G3m allele butterfly’ representation) [8]. The G1m, G2m and G3m alleles themselves, encoded by alleles of the closely linked IGHG genes, are inherited in fixed combinations or Gm haplotypes [7], [8].

The Gm polymorphism has been extensively studied in human populations, showing that a limited number of haplotypes are observed worldwide, with high frequency variations among populations from different continents [8], [13]. To date, the classical way to determine Gm allotypes is a serological hemagglutination inhibition method [14], [15]. This qualitative method will be abandoned in a near future, principally because of depletion in the collections of monospecific anti-allotype sera, obtained in the past from blood donors, and because of the difficulty of obtaining well characterized reagent monoclonal antibodies [16]. This approach is unfortunately not able to distinguish between maternal and neonatal phenotypes. Molecular techniques implying either polymerase chain reaction (PCR)-restriction fragment length polymorphism (RFLP) [17] or extensive gene sequencing [12] allow discrimination between sequences coding for the various allotypes. However these methods are not adapted to our topic of detection and quantification of neonatal antibody production.

As the sequences of the IGHG genes and alleles and their correspondence with the Gm alleles are known [8], a proteomic approach aimed at measuring peptides containing discriminatory amino acids by mass spectrometry (MS) could therefore represent a novel methodology to determine Gm3 and IGHG3 alleles and to distinguish maternal and neonatal antibodies. The objectives were i) to determine *in silico* the peptides that could discriminate between G3m and IGHG3 alleles, ii) to purify the IgG fraction with an enrichment in IgG3, iii) to detect and quantify the discriminatory peptides by mass spectrometry and iv) to test the sensitivity of our approach.

This study is a proof-of-concept step towards using mass spectrometry to detect G3m and IGHG3 alleles and to quantify them, and to distinguish maternal and infant IgG3 in neonate plasma samples where both are physiologically present.

## Methods

### Definition of Proteotypic Peptides Specific for the Human G3m and IGHG3 Alleles

Proteotypic peptides were defined by comparing the amino acid sequences of the constant regions of the four IgG subclass heavy chains gamma1, gamma2, gamma3 and gamma4, encoded by the *Homo sapiens* IGHG1, IGHG2, IGHG3 and IGHG4 genes, respectively [3], [18] [IMGT Repertoire (Sections: Protein display, Allotypes) at IMGT®, the international ImMunoGeneTics information system® [19] (http://www.imgt.org). Sequences were virtually cleaved by AspN and trypsin proteases allowing potential miscleavage. All peptides were compared to determine those that were specific to IGHG3 and discriminatory for G3m and IGHG3 alleles [8], [10], [12], [20]. A list was defined, which comprised 32 proteotypic peptides suitable for IGHG3 polymorphism analysis using liquid chromatography (LC) matrix-assisted laser desorption/ionization (MALDI) or electrospray ionisation (ESI) tandem mass spectrometry (MS/MS) (Table 1).

**Table 1:** Mass-to-charge ratios (m/z) of thirty-two G3m and IGHG3 allele peptides after AspN and trypsin digestion.

Theoretical proteotypic peptides	Miscleavage	m/z charge state			
		+1	+2	+3	+4
#1 K. TK**PW**EEQ**Y**NSTFR. V	0	1685.79	843.40	562.60	
#2 K. TK**PR**EEQ**Y**NSTFR. V	1	1655.81	828.41	552.60	414.70
#3 K. **LR**EEQ**Y**NSTFR. V	1	1442.70	721.85	481.57	
#4 V. DGVEVHNAKTK**PW**EEQ**Y**NSTFR. V	1	2635.25	1318.13	879.08	659.56
#5 R. EEQ**Y**NSTFRVVSVLTV**L**HQ. D	1	2249.15	1125.08	750.38	563.04
#6 R. EEQ**Y**NSTFRVVSVLTV**V**HQ. D	1	2235.14	1118.07	745.71	559.54
#7 K. TK**PW**EEQ**Y**NSTFRVVSVLTV**L**HQ. D	1	2761.43	1381.22	921.14	691.11
#8 K. GFYPSDIA**V**EWES**S**GQP**E**NNY**K**. T	1	2517.12	1259.06	839.71	630.03
#9 K. GFYPSDIA**M**EWES**S**GQP**E**NNY**K**. T	1	2549.09	1275.05	850.36	638.02
#10 K. GFYPSDIA**V**EWES**S**GQPENNY**N**TTPP**M**L. D	1	3143.39	1572.20	1048.46	786.60
#11 K. GFYPSDIA**V**EWES**S**GQPENNY**N**TTPP**V**L. D	1	3128.39	1564.70	1043.46	782.85
#12 K. GFYPSDIA**V**EWES**N**GQPENNY**N**TTPP**M**L. D	1	3111.42	1556.21	1037.81	778.61
#13 S. DIA**V**EWES**S**GQPENNY**K**. T	0	3170.40	1585.70	1057.47	793.35
#14 S. DIA**M**EWES**S**GQPENNY**K**. T	0	1965.88	983.44	655.96	492.22
#15 S. DIA**V**EWES**S**GQPENNY**N**TTPP**M**L. D	0	1997.85	999.43	666.62	500.21
#16 S. DIA**V**EWES**S**GQPENNY**N**TTPP**V**L. D	0	2592.16	1296.58	864.72	648.79
#17 S. DIA**V**EWES**N**GQPENNY**N**TTPP**M**L. D	0	2577.16	1289.08	859.72	645.04
#18 K. SRWQ**Q**GN**I**FSC^c^SVMHEALHN**HY**TQK. S	1	2560.18	1280.59	854.06	640.80
#19 K. SRWQ**Q**GN**I**FSC^c^SVMHEALHN**R**. **F**	1	2619.17	1310.09	873.72	655.54
#20 K. SRWQ**Q**GN**I**FSC^c^SVMHEALHN**R**. **Y**	1	3058.42	1529.71	1020.14	765.36
#21 K. SRWQ**E**GN**V**FSC^c^SVMHEALHN**R**. **F**	1	2557.19	1279.10	853.06	640.05
#22 K. SRWQ**E**GN**I**FSC^c^SVMHEALHN**R**. **F**	1	2544.16	1272.58	848.72	636.79
#23 R. WQ**Q**GN**I**FSC^c^SVMHEALHN**HY**TQK. S	0	2558.18	1279.59	853.39	640.30
#24 R. WQ**Q**GN**I**FSC^c^SVMHEALHN**R**. **F**	0	2815.28	1408.14	939.09	704.57
#25 R. WQ**Q**GN**I**FSC^c^SVMHEALHN**R**. **Y**	0	2314.06	1157.53	772.02	579.27
#26 R. WQ**E**GN**V**FSC^c^SVMHEALHN**R**. **F**	0	2301.03	1151.02	767.68	576.01
#27 R. WQ**E**GN**I**FSC^c^SVMHEALHN**R**. **F**	0	2315.04	1158.02	772.35	579.51
#28 R. WQ**Q**GN**I**FSC^c^SVMHEALHN**HY**TQKSLSLSPGK	1	3584.72	1792.86	1195.57	896.93
#29 R. WQ**Q**GN**I**FSC^c^SVMHEALHN**RF**TQK. S	1	2818.33	1409.67	940.11	705.33
#30 R. WQ**E**GN**V**FSC^c^SVMHEALHN**RF**TQK.S	1	2805.30	1403.15	935.77	702.08
#31 R. WQ**E**GN**I**FSC^c^SVMHEALHN**RF**TQK. S	1	2819.31	1410.16	940.44	705.58
#32 R. WQ**Q**GN**I**FSC^c^SVMHEALHN**RY**TQK. S	1	2834.32	1417.66	945.44	709.33

The proteotypic peptides correspond to an enzymatic AspN and trypsin digestion of the constant region of the IG gamma3 chains encoded by the *Homo sapiens* IGHG3 gene. Masses are determined for detection on the MALDI TOF and ESI Orbitrap mass spectrometers. The methionine (M) could be oxidized (+16 Da). m/z : mass-to-charge ratio; +1, +2, +3, +4 represent the peptide charge state; amino acids in bold are implicated in the discrimination between G3m and IGHG3 alleles (detailed in Table 2); «.» : site of enzymatic cut; C^c^ : carbamidomethylated cysteine.

These peptides are discriminatory for the G3m alleles and the IGHG3 alleles [8] (Table 2). Twenty-three peptides are discriminatory for one single G3m allele. Nine of these peptides are even discriminatory for a single IGHG3 allele: peptide (#6, G3m5*) for the IGHG3*09 allele, peptides (#11, #16, #21, #26, #30, G3m24*) for the IGHG3*03 allele, and peptides (#22, #27, #31, Gm6*) for the IGHG3*13 allele. Two pairs of peptides have identical sequences (#19 and #20, and #24 and #25, respectively), each member of the pair corresponding to different G3m alleles, G3m5* (#19 and #24) and G3m21* (#20 and #25). In those cases, the detection of another peptide should be required for an unambiguous G3m assignment, e.g., #15 for G3m5* or #17 for G3m21*, in the absence of serological data. The G3m alloypes, in contrast to nonimmunogenic amino acid polymorphisms, may depend on two or even three amino acids [8] found, after enzymatic digestion, on different peptides (Table 2).

**Table 2:** Characteristics of the thirty-two proteotypic peptides for *Homo sapiens* G3m and IGHG3 alleles.

Theoretical proteotypic peptides	CH domains	Positions in CHdomains [21]	Allotype and other polymorphic amino acids [8]	IGHG3 alleles from IMGT/GENE-DB [18]	G3m alleles	
					**Simplified form ** [**8**]	**Complete description ** [**8**]
#1 K. TK**PW**EEQ**Y**NSTFR. V	CH2	79–85	P82 (nG3m21), **W83** (G3m16), Y84.3	IGHG3*18, *19	G3m16*	G3m10,11,13,15,16,27
#2 K. TK**PR**EEQ**Y**NSTFR. V	CH2	79–85	P82 (nG3m21), R83, Y84.3	IGHG3*01, *02^a^, *04, *05, *06, *07, *09, *10	G3m5*	G3m5,10,11,13,14,26,27
				IGHG3*03	G3m24*	G3m5,6,11,24,26
				IGHG3*08	Unusual^b^	G3m5,14,26,27
				IGHG3*13	G3m6*	G3m5,6,10,11,14,26,27
				IGHG3*17	G3m15*	G3m10,11,13,15,27
#3 K. **LR**EEQ**Y**NSTFR. V	CH2	81–85	**L82** (G3m21), R83, Y84.3	IGHG3*14, *15, *16	G3m21*	G3m21,26,27,28
#4 V. DGVEVHNAKTK**PW**EEQ**Y**NSTFR. V	CH2	43–85	P82, (nG3m21), **W83** (G3m16), Y84.3	IGHG3*18, *19	G3m16*	G3m10,11,13,15,16,27
#5 R. EEQ**Y**NSTFRVVSVLTV**L**HQ. D	CH2	83–95	R83, Y84.3, L92	IGHG3*01, *02^a^, *04, *05, *06, *07, *10	G3m5*	G3m5,10,11,13,14,26,27
				IGHG3*03	G3m24*	G3m5,6,11,24,26
				IGHG3*08	Unusual^b^	G3m5,14,26,27
				IGHG3*13	G3m6*	G3m5,6,10,11,14,26,27
				IGHG3*17	G3m15*	G3m10,11,13,15,27
				IGHG3*14, *15, *16	G3m21*	G3m21,26,27,28
#6 R. EEQ**Y**NSTFRVVSVLTV**V**HQ. D	CH2	83–95	R83, Y84.3, V92	IGHG3*09	G3m5*	G3m5,10,11,13,14,26,27
#7 K. TK**PW**EEQ**Y**NSTFRVVSVLTV**L**HQ. D	CH2	79–95	P82, **W83** (G3m16), Y84.3, L92	IGHG3*18, *19	G3m16*	G3m10,11,13,15,16,27
#8 K. GFYPSDIA**V**EWES**S**GQPENNY**K**. T	CH3	26–80	V39, **S44** (G3m11), K79	IGHG3*06, *07	G3m5*	G3m5,10,11,13,14,26,27
				IGHG3*13	G3m6*	G3m5,6,10,11,14,26,27
#9 K. GFYPSDIA**M**EWES**S**GQPENNY**K**. T	CH3	26–80	**M39** (G3m15^d^), **S44** (G3m11), K79	IGHG3*17	G3m15*	G3m10,11,13,15,27
				IGHG3*18, *19	G3m16*	G3m10,11,13,15,16,27
#10 K. GFYPSDIA**V**EWES**S**GQPENNY**N**TTPP**M**L. D	CH3	26–84.2	V39, **S44** (G3m11), N79, **M84**	IGHG3*01, *04, *05, *09, *10, *11^c^, *12^c^	G3m5*	G3m5,10,11,13,14,26,27
#11 K. GFYPSDIA**V**EWES**S**GQPENNY**N**TTPP**V**L. D	CH3	26–84.2	V39, **S44** (G3m11), N79, V84	IGHG3*03	G3m24*	G3m5,6,11,24,26
#12 K. GFYPSDIA**V**EWES**N**GQPENNY**N**TTPP**M**L. D	CH3	26–84.2	V39, N44 (nG3m11), N79, M84	IGHG3*08	Unusual^b^	G3m5,14,26,27
				IGHG3*14, *16	G3m21*	G3m21,26,27,28
#13 S. DIA**V**EWES**S**GQPENNY**K**. T	CH3	33–80	V39, **S44** (G3m11), K79	IGHG3*06, *07	G3m5*	G3m5,10,11,13,14,26,27
				IGHG3*13	G3m6*	G3m5,6,10,11,14,26,27
#14 S. DIA**M**EWES**S**GQPENNY**K**. T	CH3	33–80	**M39** (G3m15^d^), **S44** (G3m11), K79	IGHG3*17	G3m15*	G3m10,11,13,15,27
				IGHG3*18, *19	G3m16*	G3m10,11,13,15,16,27
#15 S. DIA**V**EWES**S**GQPENNY**N**TTPP**M**L. D	CH3	33–84.2	V39, **S44** (G3m11), N79, **M84**	IGHG3*01, *04, *05, *09, *10, *11^c^, *12^c^	G3m5*	G3m5,10,11,13,14,26,27
#16 S. DIA**V**EWES**S**GQPENNY**N**TTPP**V**L. D	CH3	33–84.2	V39, **S44** (G3m11), N79, V84	IGHG3*03	G3m24*	G3m5,6,11,24,26
#17 S. DIA**V**EWES**N**GQPENNY**N**TTPP**M**L. D	CH3	33–84.2	V39, N44 (nG3m11), N79, **M84**	IGHG3*08	Unusual^b^	G3m5,14,26,27
				IGHG3*14, *16	G3m21*	G3m21,26,27,28
#18 K. SRWQ**Q**GN**I**FSCSVMHEALHN**HY**TQK. S	CH3	93–120	**Q98** (G3m13^e^), **I101** (G3m27, G3m10^f^), **H115**+ **Y116** (nG3m5, G3m15^g^)	IGHG3*17	G3m15*	G3m10,11,13,15,27
				IGHG3*18, *19	G3m16*	G3m10,11,13,15,16,27
#19 K. SRWQ**Q**GN**I**FSCSVMHEALHN**R**. **F**	CH3	93–116	**Q98** (G3m13^e^), **I101** (G3m27, G3m10^f^), **R115** (G3m26) + ***F116*** * (G3m5, G3m14* h *)*	IGHG3*01, *04, *05, *06, *07, *09, *10, *11^c^, *12^c^	G3m5*	G3m5,10,11,13,14,26,27
				IGHG3*08	Unusual^b^	G3m5,14,26,27
#20 K. SRWQ**Q**GN**I**FSCSVMHEALHN**R**. **Y**	CH3	93–116	Q98, **I101** (G3m27), **R115** (G3m26) + ***Y116*** * (G3m28)*	IGHG3*14, *15, *16	G3m21*	G3m21,26,27,28
#21 K. SRWQ**E**GN**V**FSCSVMHEALHN**R**. **F**	CH3	93–116	**E98** (G3m6^i^), **V101** (G3m24^j^), **R115** (G3m26) + **F116** (G3m5)	IGHG3*03	G3m*24	G3m5,6,11,24,26
#22 K. SRWQ**E**GN**I**FSCSVMHEALHN**R**. **F**	CH3	93–116	**E98** (G3m6^i^), **I101** (G3m27, G3m10^f^), **R115** (G3m26) + **F116** (G3m5, G3m14^h^)	IGHG3*13	G3m6*	G3m5,6,10,11,14,26,27
#23 R. WQ**Q**GN**I**FSCSVMHEALHN**HY**TQK. S	CH3	95–120	**Q98** (G3m13^e^), **I101** (G3m27, G3m10^f^), **H115**+ **Y116** (nG3m5, G3m15^g^)	IGHG3*17	G3m15*	G3m10,11,13,15,27
				IGHG3*18, *19	G3m16*	G3m10,11,13,15,16,27
#24 R. WQ**Q**GN**I**FSCSVMHEALHN**R**. **F**	CH3	95–116	**Q98** (G3m13^e^), **I101** (G3m27, G3m10^f^), **R115** (G3m26) + ***F116*** * (G3m5, G3m14* h *)*	IGHG3*01, *04, *05, *06, *07, *09, *10, *11^c^, *12^c^	G3m5*	G3m5,10,11,13,14,26,27
				IGHG3*08	Unusual^b^	G3m5,14,26,27
#25 R. WQ**Q**GN**I**FSCSVMHEALHN**R**. **Y**	CH3	95–116	Q98, **I101** (G3m27), **R115** (G3m26) + ***Y116*** * (G3m28)*	IGHG3*14, *15, *16	G3m21*	G3m21,26,27,28
#26 R. WQ**E**GN**V**FSCSVMHEALHN**R**. **F**	CH3	95–116	**E98** (G3m6^i^), **V101** (G3m24^j^), **R115** (G3m26) + **F116** (G3m5)	IGHG3*03	G3m24*	G3m5,6,11,24,26
#27 R. WQ**E**GN**I**FSCSVMHEALHN**R**. **F**	CH3	95–116	**E98** (G3m6^i^), **I101** (G3m27, G3m10^f^), **R115** (G3m26) + **F116** (G3m5, G3m14^h^)	IGHG3*13	G3m6*	G3m5,6,10,11,14,26,27
#28 R. WQ**Q**GN**I**FSCSVMHEALHN**HY**TQKSLSLSPGK	CH3	95–130	**Q98** (G3m13^e^), **I101** (G3m27, G3m10^f^), **H115**+ **Y116** (nG3m5, G3m15^g^)	IGHG3*17	G3m15*	G3m10,11,13,15,27
				IGHG3*18, *19	G3m16*	G3m10,11,13,15,16,27
#29 R. WQ**Q**GN**I**FSCSVMHEALHN**RF**TQK. S	CH3	95–120	**Q98** (G3m13^e^), **I101** (G3m27, G3m10^f^), **R115** (G3m26) + **F116** (G3m5, G3m14^h^)	IGHG3*01, *04, *05, *06, *07, *09, *10, *11^c^, *12^c^	G3m5*	G3m5,10,11,13,14,26,27
				IGHG3*08	Unusual^b^	G3m5,14,26,27
#30 R. WQ**E**GN**V**FSCSVMHEALHN**RF**TQK.S	CH3	95–120	**E98** (G3m6^i^), **V101** (G3m24^j^), **R115** (G3m26) + **F116** (G3m5)	IGHG3*03	G3m*24	G3m5,6,11,24,26
#31 R. WQ**E**GN**I**FSCSVMHEALHN**RF**TQK. S	CH3	95–120	**E98** (G3m6^i^), **I101** (G3m27, G3m10^f^), **R115** (G3m26) + **F116** (G3m5, G3m14^h^)	IGHG3*13	G3m6*	G3m5,6,10,11,14,26,27
#32 R. WQ**Q**GN**I**FSCSVMHEALHN**RY**TQK. S	CH3	95–120	Q98, **I101** (G3m27), **R115** (G3m26) + **Y116** (G3m28)	IGHG3*14, *15, *16	G3m21*	G3m21,26,27,28

The proteotypic peptides correspond to an enzymatic AspN and trypsin digestion of the constant region of the IG gamma3 chains encoded by the *Homo sapiens* IGHG3 gene.

a Partial.

b Unusual G3m allele [8], [12]. This corresponds to the IGHG3*08 allele. Allotypes G3m10, G3m11 and G3m13 are not expressed owing to the presence of CH3 Asn N44, instead of the CH3 Ser S44 usually present in the other G3m5* alleles [8].

c The IGHG3*11 and IGHG3*12 alleles differ by the number of hinge exons, 4 and 3, respectively (IMGT Repertoire, Gene table http://www.imgt.org) [8], [18].

d Expression of the allotype G3m15 is dependent, in addition to CH3 Met M39, on the presence of CH3 His H115 and Tyr Y116 [8].

e Expression of the allotype G3m13 is dependent, in addition to CH3 Gln Q98, on the presence of CH3 Ser 44 [8].

f Expression of the allotype G3m10 is dependent, in addition to CH3 Ile I101, on the presence of CH3 Ser 44 [8].

g Expression of the allotype G3m15 is dependent, in addition to CH3 His H115 and Tyr Y116, on the presence of CH3 Met M39 [8].

h Expression of the allotype G3m14 is dependent, in addition to CH3 Arg R115 and Phe F116, on the presence of CH3 Met M84 [8].

i Expression of the allotype G3m6 is dependent, in addition to CH3 Glu E98, on the presence of CH3 Ser S44 [8].

j Expression of the allotype G3m24 is dependent, in addition to CH3 Val V101, on the presence of CH3 Ser S44 [8].

Amino acids in bold are implicated in the discrimination between IGHG3 alleles. “.” : site of enzymatic cut.

Amino acids characteristic of the G3m allotypes and IGHG3 alleles are from reference [8]. They are illustrated in the ‘IMGT G3m allele butterfly’ representation [8]. Amino acid sequences are available in the IMGT Repertoire (http://www.imgt.org), IMGT/DomainDisplay and IMGT/GENE-DB [18]. Positions in the CH domains are according to the IMGT unique numbering for C domain [21].

Given the data complexity but also the richness of information (detailed in [8]), some peptides detected in this work are briefly presented. The peptide DIAVEWES**S**GQPENNYNTTPPML (#15, Gm5*) is unambiguously discriminatory for the G3m5* (G3m5,10,11,13,14,26,27) allele, as it contains the amino acid characteristic for the G3m11 allotype: IGHG3 CH3 S44 (in bold in the peptide). In contrast, as mentioned above, to be discriminatory for the G3m5* allele, the peptides SRWQ**Q**GN**I**FSCSVMHEALHN**R** (#19) and WQ**Q**GN**I**FSCSVMHEALHN**R** (#24) need to be detected with #15. Indeed they contain the amino acids characteristic for the G3m10,13,26,27 allotypes: IGHG3 CH3 Q98 (in bold) which, associated to S44 in the complete protein (and present in #15 and required), corresponds to G3m13, IGHG3 CH3 R115 (in bold) which corresponds to G3m26, and IGHG3 CH3 I101 (in bold) which corresponds to G3m27 and, associated to S44 (see above #15), to G3m10 [8] (positions are according to IMGT unique numbering for C domain [21]) (Table 2). In contrast, the peptide WQ**E**GN**V**FSCSVMHEALHN**R** (#26) is highly discriminatory for the G3m24* (G3m5,6,11,24,26) allele, as it has a unique sequence and contains the amino acids characteristic for the G3m6,24 allotypes: IGHG3 CH3 E98 (in bold) which, associated to S44 in the complete protein (and present in #16, but detection was not compulsory), corresponds to G3m6; and IGHG3 CH3 V101 (in bold) which, associated to S44 (see above #16), corresponds to G3m24 [8]. Similarly, the peptide WQ**E**GN**I**FSCSVMHEALHN**R** (#27) is highly discriminatory for the G3m6* (G3m5,6,10,11,14,26,27) allele, as it contains the amino acids characteristic of the G3m6,27 allotypes: IGHG3 CH3 E98 (in bold) which, associated to S44 in the complete protein (and present in #13, but detection was not compulsory), corresponds to G3m6; and IGHG3 CH3 I101 (in bold) which corresponds to G3m27 and, associated to S44 (see above #13), to G3m10 [8].

Table 2 shows that among the 9 peptides that are discriminatory for more than one G3m allele, 5 peptides (#9, #14, #18, #23 and #28) correspond to 2 G3m alleles and 3 IGHG3 alleles [Gm15* (IGHG3*17), and Gm16* (IGHG3*18 and *19)], 2 peptides (#8, #13) correspond to 2 G3m alleles and 3 IGHG3 alleles [G3m5* (IGHG3*06 and *07) and G3m6* (IGHG3*13)] whereas only 2 peptides are not discriminatory, being found in 4 (#2) and 5 (#5) different G3m alleles.

### Plasma Samples Collection

Plasma samples from ten individuals were analyzed. One blood sample was collected in France from a healthy adult volunteer (EUA1: EUropean Adult 1) who gave her written informed consent for her blood to be used for the purpose of the present study. Plasma samples from nine African individuals were obtained from two studies performed on human genetic determinants of malaria in the south of Benin by the UMR 216 team, and for which ethical clearance was obtained. Four of these samples originated from a study conducted in 2006–2007 among 155 schoolchildren (BEC1 to BEC4: BEninese Children 1 to 4) belonging mainly to the Fon ethnic group [22]. Acceptance of the study was first obtained near the coordinating doctor of the sanitary zone and the inspector of education. Oral information on the study was thereafter provided by the research team to the school director and the teachers before being dispensed to the members of the association of schoolchildren’s parents. A collective written informed consent was obtained from the head/person in charge of the association of parents, which took into account their individual positions. The study was authorized by the institutional “Ethics Committee of the Faculté des Sciences de la Santé” (FSS) from the Université d’Abomey-Calavi (UAC) in Benin. The five other plasma samples concerned a mother and child pair (BEM1: BEninese Mother 1 and BEI1: BEninese Infant 1) as well as three other mothers (BEM2 to BEM4) issued from a malaria birth cohort of 627 neonates and their mothers who were followed-up from 2007 to 2010 in a semi-rural area [23]. At delivery, maternal peripheral blood and infant cord blood were drawn. Thereafter, the blood of child BEI1 was collected quarterly until he reached eighteen months. Plasma samples at 3, 6 and 9 months of age were selected for the purpose of the study. Upon arrival at the maternity clinic for delivery, women were given information on the study protocol. The informed consent written in French and in Fon was then submitted for approval. The protocol was approved by both the institutional “Ethics Committee of the Faculté des Sciences de la Santé” (FSS) in Benin and the IRD “Consultative Committee on Professional Conduct and Ethics” (CCDE) in France.

In all cases, blood was collected into 5 mL EDTA Vacutainer^®^ tubes and after centrifugation, plasma samples were frozen at −20°C. One milliliter of fresh plasma from the European individual was also analyzed by comparison with the frozen one.

### Serological Determination of Gm Allotypes

Gm allotypes in plasma samples were analyzed by a qualitative standard hemagglutination inhibition method [14], [15]. In brief, human blood group O Rh+ erythrocytes were coated with anti-Rh antibodies of known Gm allotypes. Plasma sample and reagent monospecific anti-allotype antibody were added. Plasma containing IgG with a particular Gm allotype inhibited hemagglutination by the corresponding reagent anti-allotype antibody, whereas plasma sample that was negative for this allotype did not.

Concerning the infant BEI1, allotype determination was assessed at 15 months of age to avoid any residual presence of maternal antibodies [23].

### Total IgG3 Purification

Fresh or frozen (at −20°C) plasma samples were tested in parallel in order to evaluate potential differences in the final result. Frozen plasma samples were used after centrifugation in order to eliminate the fibrin aggregates. A quantity of 250 µL of plasma was sufficient to detect and quantify allotype peptides from purified total IgG3.

First, a Protein A column (HiTrap Protein A HP, GE Healthcare, France) was used according to the manufacturer’s instructions. This column is intended to retain IgG1, IgG2 and IgG4 leaving IgG3 in the filtrate. Efficient binding of IgG1, IgG2 and IgG4 to the Protein A column necessitates low ionic strength, a pH value of 7. The flow-through fraction was injected in a Protein G column (Protein G Sepharose HP SpinTrap, GE Healthcare, France) which presents high affinity for the Fc fragment of IgG from a large variety of species including human IgG3. Antibody binding was performed at neutral pH and elution was obtained by lowering the pH. The eluted material was neutralized to preserve the integrity of acid-labile IgG.

### Validation of IgG3 Purification

Validation of IgG3 purification was tested on several samples. All filtrate and elution fractions from Protein A and Protein G affinity chromatography were either migrated on a 12% Sodium Dodecyl Sulfate PolyAcrylamide Gel Electrophoresis (SDS-PAGE) or assayed in an Enzyme-Linked ImmunoSorbent Assay (ELISA) in order to measure the quantities of IgG3 all along the purification process. SDS-PAGE was performed on the EUA1 fractions and ELISA on BEC3 and BEC4 fractions.

Briefly, for the SDS-PAGE, 15 µL of a 2X β-mercaptoethanol/Laemmli buffer were added to 15 µL of filtrate or elution fractions. After boiling at 100°C for 5 minutes, samples were migrated on a 12% SDS-PAGE. Gels were colored with colloidal Coomassie Blue. For the ELISA, a total of 100 µL of a Phosphate Buffered Saline (PBS) solution containing 0.1 µg of a mouse monoclonal anti-human IgG3 (clone ZG4, gift from P. Aucouturier) were coated in a 96-well plate (Thermo Fisher Scientific, San José, CA) overnight at 4°C. Wells were blocked with 150 µL of PBS added with 4% Bovine Serum Albumin (BSA). After 4 washes, each fraction diluted 1/100 000 was incubated 1 hour at 37°C. Controls were constituted with a pool of European sera. A human monoclonal purified IgG3 (Binding Site, UK) was used as standard. A horseradish peroxidase (HRP)-conjugate goat anti-human IgG (Sigma Aldrich, France) was incubated for 1 hour at room temperature. Reading was done at 420 nm 10 minutes after addition of 3,3′,5,5′-tetramethylbenzidine (Microwell Peroxidase substrate, KPL, USA).

### Reduction, Alkylation and Enzymatic Digestion

Dithiothreitol (DTT, 20 mM) was added to 37 µL of purified samples for 30 minutes at 56°C in order to reduce disulfide bonds. Except for SRM experiments, chloroacetamide (final concentration 25 mM) was added for 30 minutes at room temperature for protecting thiol groups. AspN (Roche Diagnostics, France, 10 ng/µL) was added before incubation for 3 hours at 37°C or overnight at 30°C. The same incubation conditions were then applied to trypsine (Promega, France, 10 ng/µL). The enzymatic digestion was stopped with trifluoroacetic acid (TFA, Pierce, France, 0.5% final).

Purified samples from either fresh or frozen plasma samples show no difference in the final results. The treatment of IgG3 purified samples with GluC or with papaïne before AspN and trypsin digestion was tested on several samples but did not bring improvements in the final results.

### Mass Spectrometry (MS) Analysis

#### Nano-liquid chromatography (nLC) MALDI time-of-flight (TOF) MS and tandem mass spectrometry (MS/MS)

Peptides were concentrated and separated by nano High-Performance Liquid Chromatography (nHPLC) using an Ultimate® 3000 (Dionex, Netherlands). Briefly, 10 µl of elution fraction were injected and trapped using solvent A (TFA 0.1%, acetonitrile (ACN, Carlo-Erba, France) 2%) at a 30 µL/minute loading flow rate for 3 min in a C18 trap column (C18 PepMap, 5 µm, 100 Å, 300 µm i.d., 5 mm length) and separated in the analytical column (C18 PepMap, 3 µm, 100 Å, 75 µm i.d., 15 cm length) with a discontinuous gradient from 7% solvent B (ACN 80%, solvent A 20%) to 20% in 7 min and from 20% to 60% in 58 min at 300 nL/min. A Probot (Dionex, France) fraction collector was used to spot 192 collected fractions on a MALDI target (ion source). Spotted fractions were mixed 1∶9 (v/v) with 2 mg/mL of alpha-cyano-4-hydroxycinnamic acid (HCCA, Laser Biolabs) in ACN 70% TFA 0.1% containing 3 fmoles/spot of a standard peptide (Glu fibrinopeptide) and analyzed by time-of-flight (TOF) (mass analyzer), using a 4800 MALDI TOF/TOF™ analyzer (Applied Biosystems, France).

Spectra acquisition and processing were performed using the 4000 Series Explorer™ software (Applied Biosystems, France) version 3.5 in positive reflectron mode at fixed Laser fluency with low mass gate and delayed extraction. External plate calibration was performed using 4 calibration points spotted throughout the plate, and internal calibration using Glu-fibrinopeptide yielded under 20 ppm accuracy. For each MALDI spot, 500 spectra (10 sets of 50 averaged Laser shots) were acquired and summed in the 700 to 4000 m/z range. The data above signal-to-noise (s/n) ratio of 20 were filtered before deisotoping. In each MALDI spot, the 8 most abundant peaks (minimum s/n 20) were selected for fragmentation by collision-induced dissociation (CID) starting with the least abundant. Neighboring precursors within resolution of 200 were excluded. One thousand MS/MS spectra per precursor were summed by increments of 50.

Processing included baseline subtraction and Stavitsky Golay smoothing with 3 points across peak and a polynomial order of 4. Peak lists reflect monoisotopic values from isotope clusters with an s/n ratio of minimum 22. Generated MS/MS peak lists were subsequently submitted to an in-house Mascot (Matrix science) version 2.2 search engine [24] to identify peptides (detailed in the “Database searching” paragraph).

#### nLC Linear Trap Quadrupole (LTQ) ESI Orbitrap MS and MS/MS

A nanoHPLC system Ultimate® 3000 Rapid Separation Liquid Chromatographic (RSLC) (Dionex, Netherlands) was coupled to an Orbitrap Velos (Thermo Fisher Scientific, San José, CA), hybrid mass spectrometer that combines a LTQ ion trap in ESI mode and an Orbitrap mass analyzer technology. Briefly, peptides were desalted on a C18 reverse phase precolumn (C18 PepMap, 3 µm, 100 Å, 75 µm i.d., 2 cm length) using a loading buffer containing H_2_O/ACN/TFA 98∶2∶0.1 (v/v) at 5 µL/min. Peptides were then separated on a C18 reverse phase analytical column (C18 PepMap, 2 µm, 100 Å, 75 µm i.d., 15 cm length) with a 45 min gradient from 100% A (H_2_O/ACN/formic acid 95∶5∶0.1 (v/v/v)) to 40% B (H_2_O/ACN/formic acid 20∶80∶0.085 (v/v/v)).

Data dependent acquisition with the Orbitrap Velos was done throughout the elution process: 1 full scan MS was followed by up to 10 LTQ MS/MS CID spectra on the most abundant precursors detected in the MS scan with a dynamic exclusion of 24 seconds for previously fragmented precursors. Mass spectrometer settings were: full MS (Automatic Gain Control (AGC): 1E6, resolution: 3E4, m/z range 400–2000, maximum ion injection time: 1000 ms); MS/MS (AGC: 1E4, maximum injection time: 200 ms, minimum signal threshold: 2000, isolation width: 2 Da). The fragmentation was permitted for precursors with a charge state of 2, 3 or 4.

The Progenesis LC-MS software (Version 3.0; Nonlinear Dynamics Ltd) for label-free semi-quantitative data analysis was used to quantify the variation of discriminatory peptides on the basis of retention time, m/z and peak intensity (peak area) on samples. Progenesis software processed the raw data files in two steps: alignment followed by normalization. The data file that yielded most features (ratio 1∶1) was used as reference to align retention time in all other measurements. Correction for experimental variations was done by calculating the robust distribution of all ratios (log (ratio)). The peaks (the features) were converted into intensity lists by using the raw data files. The data were filtered using the following criteria: features with masses between 300 and 1700 Da, retention time 8–25 minutes and charge state 2 to 4. A matrix of all samples was generated, consisting of all masses with corresponding peak intensities of every sample. A resulting.mgf file was exported from Progenesis and imported in Mascot software, interrogation was performed on a home-made database (detailed in the “Database searching” paragraph). A resulting XML file was then imported in Progenesis to assign peptides to features. The results of normalized peptide abundances were analyzed using Excel software.

#### nLC Selected Reaction Monitoring (SRM) on Triple quadrupole mass spectrometry

An Ultimate® 3000 RSLC (Dionex, Netherlands) coupled to a TSQ Vantage™ mass spectrometer (Thermo Fisher Scientific, San José, CA) in ESI mode. Briefly, peptides were loaded and washed on a C18 reverse phase precolumn (C18 PepMap, 3 µm, 100 Å, 75 µm i.d., 2 cm length) using a loading buffer containing H_2_O/CAN/TFA 98∶2∶0.05 (v/v/v) at 6 µL/min. Peptides were then separated on a C18 reverse phase analytical column (C18 PepMap, 2 µm, 100 Å, 75 µm i.d., 15 cm length) with a 60 min gradient from 99% A (H2O/ACN/formic acid 98∶2∶0.1 (v/v/v)) to 50% B (H2O/ACN/formic acid 10∶90∶0.1 (v/v/v)) at 300 nL/min. All the data were acquired in triplicate and blank runs were interposed until necessary to avoid peptide carry-over effects. SRM acquisitions were performed in scheduled mode. The SRM transitions (precursor/product pairs) were recorded at the retention time +/−4 min as measured during the optimization step. In the most complex part of the chromatogram, where transitions overlap the most, the dwell time associated to the SRM method is less than 2s. The first and third quadrupole were set to 0.7 Da peak width and the transitions were chosen as detailed in Table 3 [25]. For all the transitions related to the WQ**Q**GN**I**FSCSVMHEALHN**R** (#24 G3m5*) and WQ**E**GN**V**FSCSVMHEALHN**R** (#26 G3m24*) peptides dwell time of 100 ms was used. The sensitivity threshold tested with AQUA™ peptides (Thermo Fisher, Germany) was 10 fmol of injected peptide. AQUA™ peptides are synthetic peptides, chemically identical with the same structure but isotopically modified using incorporating heavy and stable isotopes (13C or 15N) spiked in the biological sample in order to serve as internal standards for chromatographic normalization (retention time and peak area).SRM data processing and absolute quantitation using AQUA™ peptides were performed by Pinpoint version 1.2 (Thermo Fisher, Germany).

**Table 3:** Transitions used in SRM experiments for peptides WQQGNIFSCSVMHEALHNR (#24, G3m5*) and WQEGNVFSCSVMHEALHNR (#26, G3m24*).

Sequence	Precursor 3+ (m/z)	Fragment +1 (m/z)	Fragment type
WQQGNIFSCSVMHEALHNR	753.0	289.1	y2
WQQGNIFSCSVMHEALHNR	753.0	315.1	b2
WQQGNIFSCSVMHEALHNR	753.0	426.1	y3
WQQGNIFSCSVMHEALHNR	753.0	610.3	y5
WQQGNIFSCSVMHEALHNR	753.0	876.4	y7
WQQGNIFSCSVMHEALHNR	753.0	1007.4	y8
WQ**Q**GN**I**FSCSVMHEALHN**R**	756.3	299.1	y2
WQQGNIFSCSVMHEALHN**R**	756.3	315.1	b2
WQQGNIFSCSVMHEALHN**R**	756.3	436.1	y3
WQQGNIFSCSVMHEALHN**R**	756.3	620.3	y5
WQQGNIFSCSVMHEALHN**R**	756.3	886.4	y7
WQQGNIFSCSVMHEALHN**R**	756.3	1017.5	y8
WQQGNIFSCSVM(ox)HEALHNR	758.3	289.1	y2
WQQGNIFSCSVM(ox)HEALHNR	758.3	315.1	b2
WQQGNIFSCSVM(ox)HEALHNR	758.3	426.1	y3
WQQGNIFSCSVM(ox)HEALHNR	758.3	610.3	y5
WQQGNIFSCSVM(ox)HEALHNR	758.3	876.4	y7
WQQGNIFSCSVM(ox)HEALHNR	758.3	1023.5	y8
WQQGNIFSCSVM(ox)HEALHN**R**	761.7	299.1	y2
WQQGNIFSCSVM(ox)HEALHN**R**	761.7	315.1	b2
WQQGNIFSCSVM(ox)HEALHN**R**	761.7	436.1	y3
WQQGNIFSCSVM(ox)HEALHN**R**	761.7	620.3	y5
WQQGNIFSCSVM(ox)HEALHN**R**	761.7	886.4	y7
WQQGNIFSCSVM(ox)HEALHN**R**	761.7	1033.5	y8
WQEGNVFSCSVMHEALHNR	748.7	289.1	y2
WQEGNVFSCSVMHEALHNR	748.7	315.1	b2
WQEGNVFSCSVMHEALHNR	748.7	426.1	y3
WQEGNVFSCSVMHEALHNR	748.7	610.3	y5
WQEGNVFSCSVMHEALHNR	748.7	876.4	y7
WQEGNVFSCSVMHEALHNR	748.7	1007.4	y8
WQEGNVFSCSVMHEALHN**R**	752.0	299.1	y2
WQEGNVFSCSVMHEALHN**R**	752.0	315.1	b2
WQEGNVFSCSVMHEALHN**R**	752.0	436.1	y3
WQEGNVFSCSVMHEALHN**R**	752.0	620.3	y5
WQEGNVFSCSVMHEALHN**R**	752.0	886.4	y7
WQEGNVFSCSVMHEALHN**R**	752.0	1017.5	y8
WQEGNVFSCSVM(ox)HEALHNR	754.0	289.1	y2
WQEGNVFSCSVM(ox)HEALHNR	754.0	315.1	b2
WQEGNVFSCSVM(ox)HEALHNR	754.0	426.1	y3
WQEGNVFSCSVM(ox)HEALHNR	754.0	610.3	y5
WQEGNVFSCSVM(ox)HEALHNR	754.0	876.4	y7
WQEGNVFSCSVM(ox)HEALHNR	754.0	1023.5	y8
WQEGNVFSCSVM(ox)HEALHN**R**	757.3	299.1	y2
WQEGNVFSCSVM(ox)HEALHN**R**	757.3	315.1	b2
WQEGNVFSCSVM(ox)HEALHN**R**	757.3	436.1	y3
WQEGNVFSCSVM(ox)HEALHN**R**	757.3	620.3	y5
WQEGNVFSCSVM(ox)HEALHN**R**	757.3	886.4	y7
WQEGNVFSCSVM(ox)HEALHN**R**	757.3	1033.5	y8

3+: The precursor ions were in triply-charged form; m/z: mass to charge ratio; ox: oxidized methionine (M), accepted nomenclature for fragment ions as proposed by Roepstorff and Fohlman [25]; bolded are the heavy arginine.

#### Database searching

MS/MS spectra originating from mass spectrometers were submitted to Mascot. The database search was a home-made concatenation of IMGT® IGHG allele database [18], [20] (IMGT Repertoire. Sections: Protein displays, Alignments of alleles, http://www.imgt.org), with the SwissProt fasta database release 2011_01, 529 942 sequences, 189 364 547 residues. The precursor mass tolerance was set to 20 or 3 ppm for MALDI MS and ESI LTQ MS respectively, and the fragment mass tolerance to 0.35 and 0.45 Da for MALDI MS/MS and ESI LTQ MS/MS, respectively. Two miscleavages and the partial oxidation of methionines were permitted. The search was not species-restricted. Selected enzymes were AspN and Trypsin. A filter was applied to the search in order to keep the probability of false positive peptide identification below 5%. A minimum Mascot score value of 25 was set for peptide selection.

## Results

### IgG3 Purification from Plasma Samples

**Figure 1:**
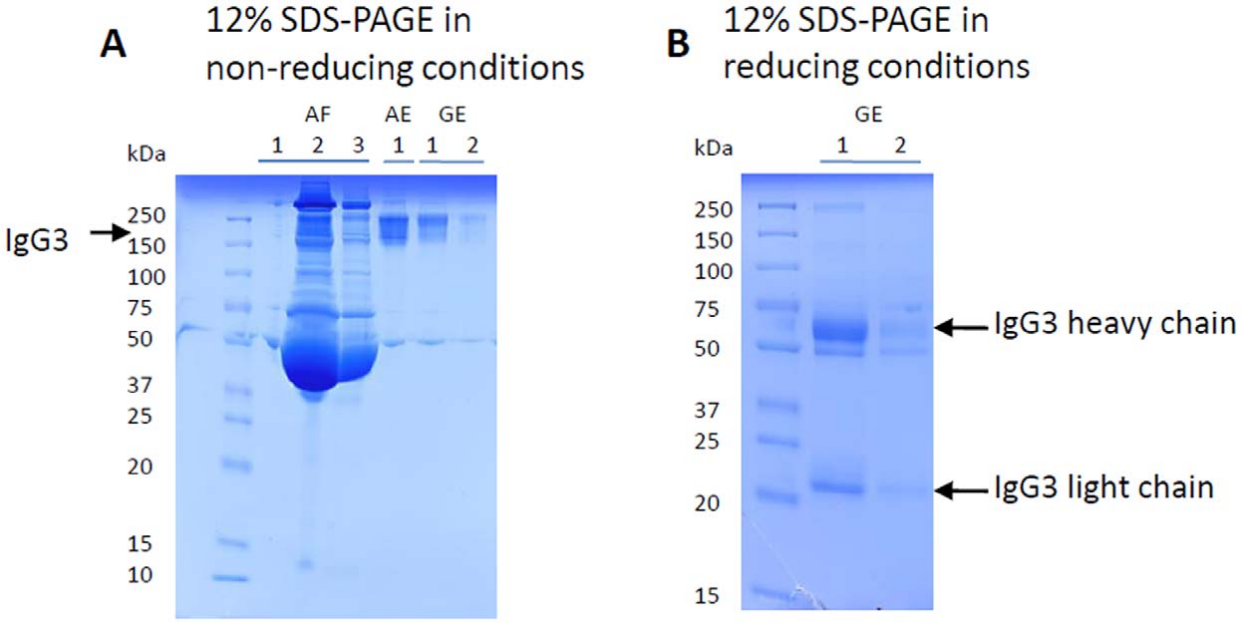
Protein-A and Protein-G purification fractions from the EUA1 plasma sample on an acrylamid gel. A. 12% SDS-PAGE in non-reducing conditions: lines AF1 to AF3: consecutive filtrate fractions of a Protein A column containing plasma proteins including IgG3; line AE: Elution fraction of a Protein A column containing IgG1, IgG2, IgG4; lines GE1 and GE2: consecutive elution fractions of a Protein G column containing IgG3. B. 12% SDS-PAGE in reducing conditions: lines GE1 and GE2: consecutive elution fractions of a Protein G column containing IgG3.

The SDS-PAGE was performed on EUA1. On the non-reducing SDS-PAGE (Figure 1A), the filtrate fractions of the Protein A column (AF) were constituted by many plasma proteins including the IgG3 with a band at 160 kDa approximately, whereas the elution fraction (AE) contained a majority of the other subclasses (IgG1, IgG2 and IgG4). The elution fraction from the Protein G column (GE) contained enriched IgG3 (22% of plasma IgG3) but also unexpected plasma proteins. On the reducing SDS-PAGE (Figure 1B), dissociated IgG3 heavy and light chains migrated at 65 kDa and 20 kDa, respectively. The measurement of IgG subclass levels by ELISA on BEC3 and BEC4 revealed an abnormal presence of IgG3 in the AE fraction (24.4% of plasma IgG3 were found in this fraction).

In conclusion, the use of Protein A and G columns allowed a final enrichment of IgG3 in GE (27% and 81%, respectively), although with a loss of IgG3 and without being exclusive of the other IgG subclasses. As the list of discriminatory peptides defined in Table 1 is specifically representative of IGHG3, the mass spectrometric analysis should not be hampered by the presence of contaminating heavy chains from IgG1, 2 and 4.

### Detection of Distinct Proteotypic G3m Allotype Peptides by Mass Spectrometry

Experiments were first done on plasma samples from three Beninese individuals known to harbor distinct G3m alleles: BEC1 homozygous for G3m5*, BEC2 heterozygous for G3m5*/G3m24* and BEM3 heterozygous G3m5*/G3m6*, and then on the other seven samples Altogether eight discriminatory peptides were detected. They included three peptides for G3m5* (#15, #19, #24), four peptides for G3m24* (#11, #21, #26, #30), and one peptide for G3m6* (#27). The (#24, G3m5*) and (#26, G3m24*) peptides were found as expected, based on the corresponding serological data of the samples, and on the results of the MS/MS, but with MALDI having missing peaks compared to Orbitrap. At this proof of concept stage, these experimental differences that do not interfere with the final results will be explored further but are not discussed here. Interestingly, the presence of unexpected peptides corresponding to three G3m alleles in a single sample may be explained by the cross-contamination of the samples.

**Figure 2:**
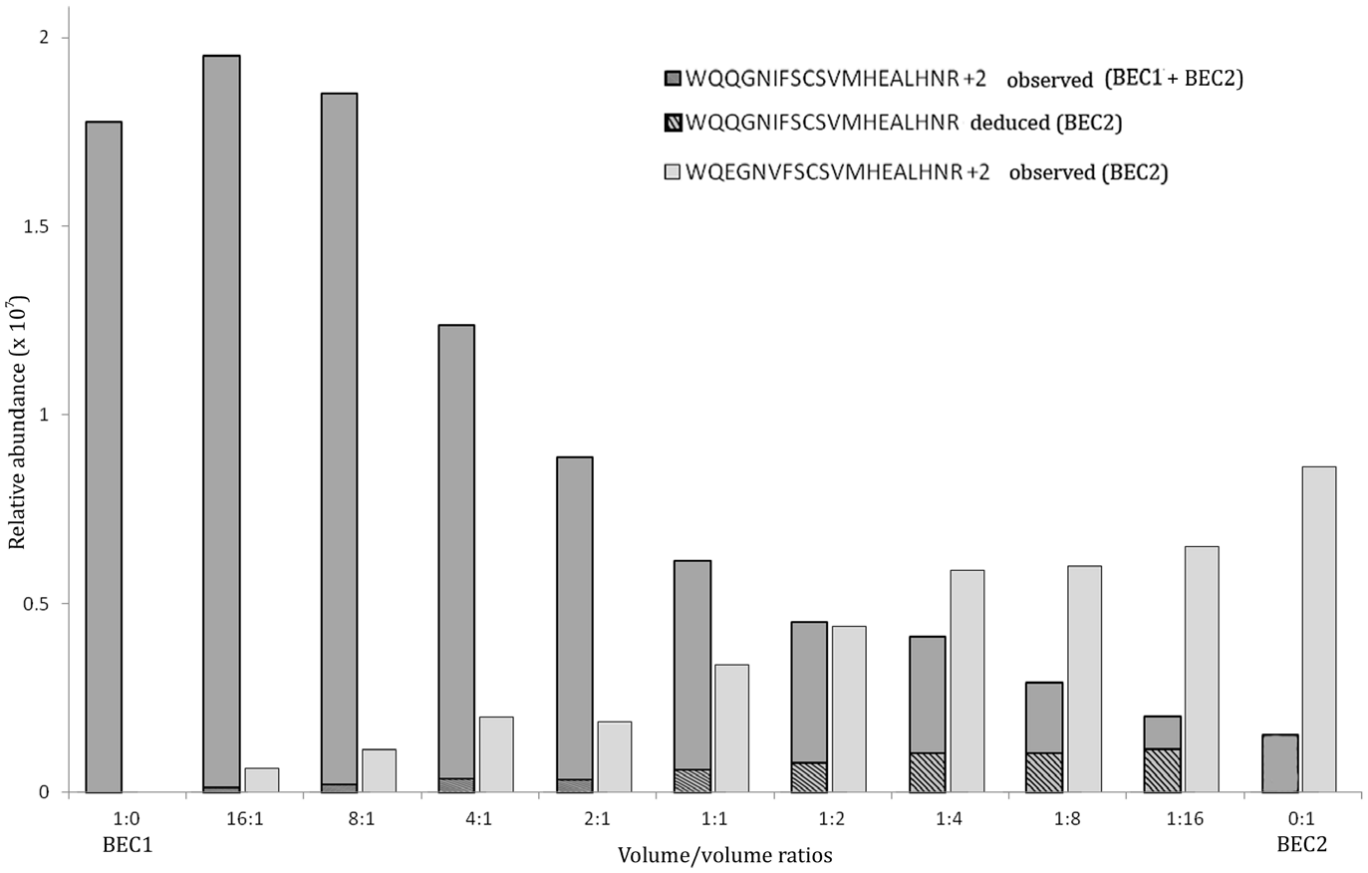
ESI MS/MS fragmentation profiles from three main proteotypic allelic peptides. Y axis represents the logarithm of the relative abundance +1, x axis represents the mass to charge ratio (m/z). A. Spectra from the sample (BEC1), fragmentation of peptide (#24, G3m5*) (IGHG3*01,*04 to*07, *09 to*12, CH3 95–116). The database search identifies the sequence 332–350 for IMGT reference AJ390260|IGHG3*15. This sequence is found in 9 IGHG3 alleles of G3m5* and is identical to peptide (#25, G3m21*) found in 3 IGHG3 alleles (of which IGHG3*15) of G3m21* (as detailed in Table 2 and discussed in 2.1). In BEC1, the parallel identification of the (#19, G3m5) peptide confirmed that indeed the fragmented peptide is (#24, G3m5*) (1+)*y* fragment ion series for m/z 1129.02 with 2 charges and free cysteine; accepted nomenclature for fragment ions as proposed by Roepstorff and Fohlman [25]. B. Spectra from the sample (BEC2), fragmentation of peptide (#26, G3m24*) (IGHG3*03, CH3 95–116), sequence 347–365 for IMGT reference X16110|IGHG3*03. In this case, the peptide (#26, G3m24*) is highly discriminatory as it corresponds not only to a single allotype (G3m24*) but also to a single IGHG3 allele (IGHG3*03), (1+)*y* fragment ion series for m/z 1122.50 with 2 charges and free cysteine. C. Spectra from the sample (BEM3), fragmentation of peptide (#27, G3m26*) (IGHG3*13, CH3 95–116), sequence 347–365 for IMGT reference AJ390244|IGHG3*13. As in (B), the peptide (#27, G3m26*) is highly discriminatory as it corresponds to a single allotype (G3m6*) and to a single IGHG3 allele (IGHG3*13), (1 or 2+)*y* or (1+)*b* fragment ion series for m/z 753.02 with 3 charges and free cysteine.

The fragmentation profiles of the three discriminatory peptides WQ**Q**GN**I**FSCSVMHEALHN**R** (#24, G3m5*), WQ**E**GN**V**FSCSVMHEALHN**R** (#26, G3m24*) and WQ**E**GN**I**FSCSVMHEALHN**R** (#27, G3m6*) detected and identified by the Orbitrap are shown in Figure 2. The peptides WQ**Q**GN**I**FSCSVMHEALHN**R** (#24, G3m5*) and WQ**E**GN**V**FSCSVMHEALHN**R** (#26, G3m24*) for which AQUA peptides were available were also detected, identified and quantified by the SRM method (Table 3).

These results demonstrate that MS allows identifying the proteotypic peptides that characterize G3m and IGHG3 alleles and therefore represents a choice method for the analysis of the IGHG3 amino acid polymorphisms.

### Application of the Mass Spectrometry Approach to a Mixture of Plasma Samples

**Figure 3:**
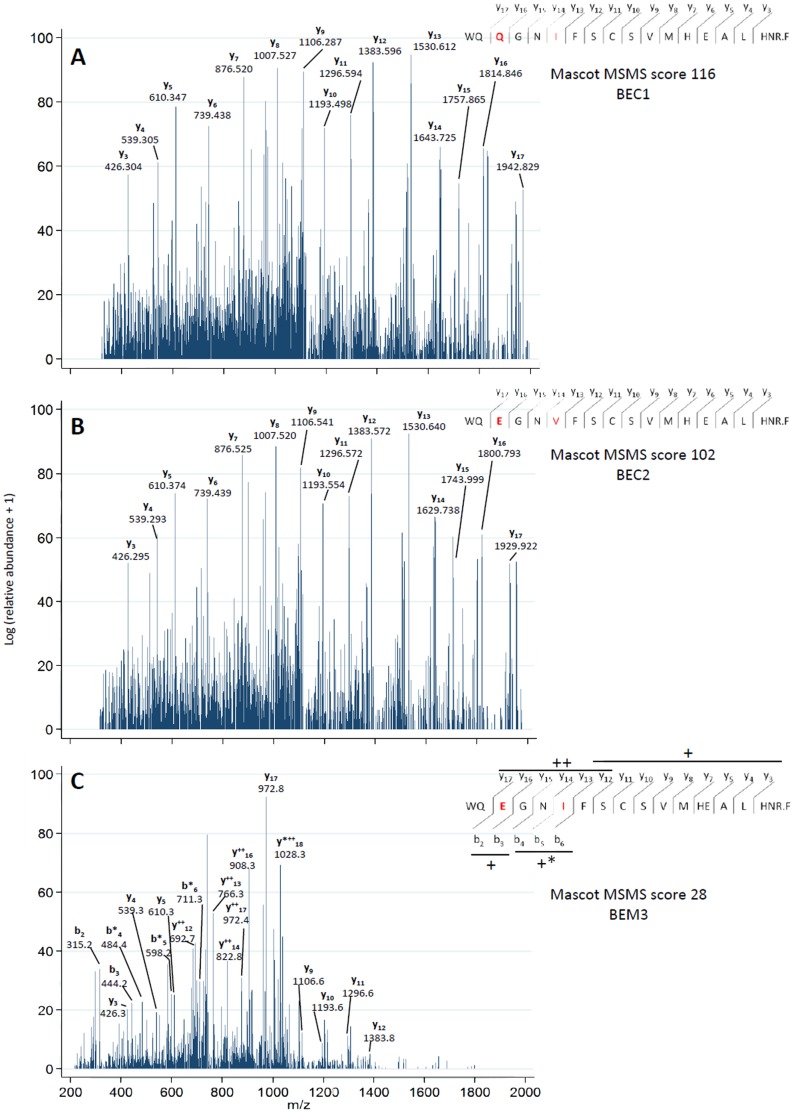
Relative abundance of a volume/volume mixture from BEC1 (G3m5*) and BEC2 (G3m5*/G3m24*) plasma samples. The relative abundance was calculated by the Progenesis LC-MS software for label-free semi-quantitative data analysis (detailed in 2.7); +2: dicharged peptides; the light grey bars represent the measure of the WQ**E**GN**V**FSCVMHEALHNR (#26, G3m24*) peptide (only brought by BEC2), the dark grey bars taken all together represent the measure of the WQ**Q**GN**I**FSCVMHEALHNR (#24, G3m5*) peptide (brought by both BEC1 and BEC2). The dark grey bars were divided into a hatched part (for the deduced signal attributable to BEC2, calculated from the 0∶1 ratio) and a non-hatched part (for the deduced signal attributable to BEC1).

In order to evaluate the sensitivity and specificity of the methodology, volume/volume ratios of 1∶0, 16∶1, 8∶1, 4∶1, 2∶1, 1∶1, 1∶2, 1∶4, 1∶8, 1∶16, 0∶1 were prepared with IgG3 purified fractions from BEC1 (homozygous for G3m5*) and BEC2 (heterozygous for G3m5*/G3m24*) samples before their reduction, alkylation and enzymatic digestion (Figure 3). As a reminder, both samples shared the WQ**Q**GN**I**FSCSVMHEALHN**R** peptide (#24, G3m5*) and BEC2 presented the additional WQ**E**GN**V**FSCSVMHEALHN**R** peptide (#26, G3m24*)**.** This experiment illustrates the artificial mixture of mother and child IgG3 in an infant plasma sample, in the case of one of them being heterozygous for 2 distinct G3m alleles and the other homozygous for one of these alleles (Figure 3). The respective signal intensities were measured by the Progenesis software.

The most diluted peptides in the 1∶16 and 16∶1 ratios were successfully detected, demonstrating the high sensitivity and specificity of the method. Furthermore the signal intensities were adequately related to the real ratios of the sample mixtures.

The 1∶0 and 0∶1 ratios presented different relative abundances that were related to their absolute quantification made by SRM. Indeed, in the BEC1 sample that is homozygous G3m5*, the concentration of WQ**Q**GN**I**FSCSVMHEALHN**R** (#24, G3m5*) was 262.2+/−13.4 fmol/µL whereas, as expected, WQ**E**GN**V**FSCSVMHEALHN**R** (#26, G3m24*) was absent. In the BEC2 sample that is heterozygous G3m5*/G3m24*, the concentrations of WQ**Q**GN**I**FSCSVMHEALHN**R** (#24, G3m5*) and WQ**E**GN**V**FSCSVMHEALHN**R** (#26, G3m24*) were 56.0+/−4.9 fmol/µL and 990.3+/−20.7 fmol/µL, respectively. The 0∶1 ratio represented the relative quantities of proteotypic peptides brought by each of the 2 polymorphic gamma3 chains of BEC2. Considering that the ratio between these 2 peptides was constant and applicable to each volume mixture, it was possible to deduce the quantity of WQ**Q**GN**I**FSCSVMHEALHN**R** (#24, G3m5*) attributable to BEC1 and BEC2, respectively (Figure 3).

### Application of the Mass Spectrometry Approach to Paired Mother and Child Plasma Samples

**Figure 4:**
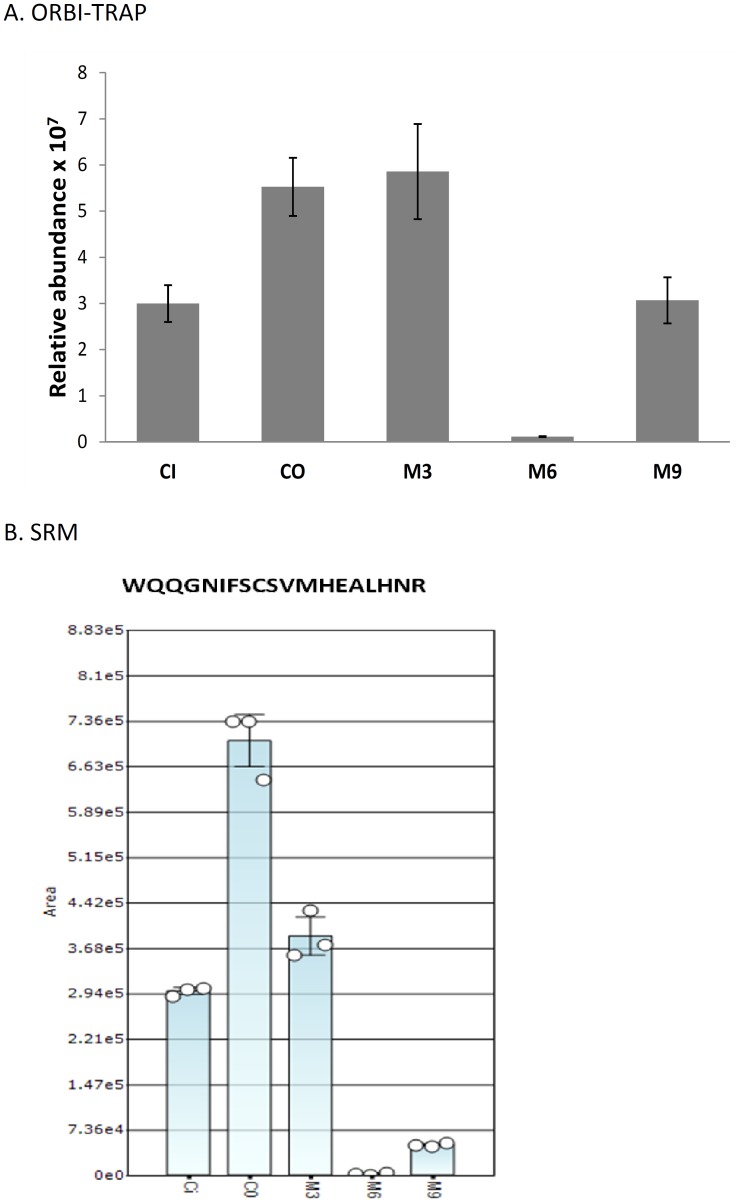
Relative abundance of peptide WQQGNIFSCSVMHEALHNR (#24, G3m5*) in a mother and her newborn plasma samples. CI = mother circulating plasma, CO = plasma from cord blood, M = Infant plasma at 3, 6 and 9 months; result based on 3 technical replicates. A. the peptide signals were measured by LTQ Orbitrap and the relative abundance was calculated by the Progenesis LC-MS software. B. the peptide signals were measured by SRM.

MS analysis was performed on total IgG3 purified from plasma samples of one Beninese mother (BEM1) and her baby (BEI1: cord blood, 3, 6 and 9 months) (Figure 4). As the mother BEM1 was serologically identified as G3m5*/G3m24* heterozygous, a combination of the peptides WQ**Q**GN**I**FSCSVMHEALHN**R** (#24, G3m5*) and WQ**E**GN**V**FSCSVMHEALHN**R** (#26, G3m24*) was expected. The child BEI1 being homozygous for G3m5*, the sole presence of the WQ**Q**GN**I**FSCSVMHEALHN**R** peptide (#24, G3m5*) was expected.

The graph in Figure 4 illustrates the decrease in the WQ**Q**GN**I**FSCSVMHEALHN**R** peptide (#24, G3m5*) between birth (CO sample) or three months of age (M3 sample) and six months of age (M6 sample) (Figure 4A). This is concomitant to the loss of maternal-transmitted IgG3 which partially comprise this peptide. At nine months of age (M9 sample), only the WQ**Q**GN**I**FSCSVMHEALHN**R** peptide (#24, G3m5*) corresponding to IgG3 neo-synthesized by the infant is visualized.

The WQ**E**GN**V**FSCSVMHEALHN**R** peptide (#26, G3m24*) was expected in the mother’s sample (CI) as well as in the samples corresponding to the first months of life of her child (CO, M3, M6), but it was not visualized in the Orbitrap. The detection threshold of these peptides seems to be too low in the sample.

Therefore, the optimized SRM strategy was used to monitor the WQ**E**GN**V**FSCSVMHEALHN**R** (#26, G3m24*) and WQ**Q**GN**I**FSCSVMHEALHN**R** (#24, G3m5*) peptides with their corresponding AQUA peptides. The WQ**Q**GN**I**FSCSVMHEALHNR (#24, G3m5*) peptide was found and quantified in agreement with the Orbitrap data (Figure 4B) but the detection of peptide WQ**E**GN**V**FSCSVMHEALHN**R** (#26, G3m24*) was not possible in this particular case of mother-child pair samples. Two hypotheses may be proposed whereby i) BEM1 could be homozygous for the G3m5* allele inversely to the results provided by the hemagglutination inhibition method or ii) the WQ**E**GN**V**FSCSVMHEALHN**R** (#26, G3m24*) signal was undetected because it was under the signal/noise ratio.

In conclusion, both quantitative methods, either relative or absolute, provided satisfactory detection of the expected peptides from the two alleles whether in the artificial mixture or in the mother/neonate sera. Protein carbamidomethylation of cysteins was skipped in SRM experiments to avoid “multiple signals” of the peptide due to incomplete reaction.

## Discussion

MS methodology for the detection and quantification of the G3m and IGHG3 alleles is described. At each step, from plasma purification up to peptide sequence analysis, the most adequate amongst different tested protocols were chosen. The IgG3 purification step is a crucial point. Affinity chromatography using successively Protein A and Protein G columns was chosen in the present study. An alternative choice would be to process plasma samples on fast protein size-exclusion liquid chromatography in order to optimize the quantity of recovered material and also to standardize the experiment thanks to an automated system. The purification yield of the protein A column was not satisfying in the present study and an improvement of the IgG purification process could consist in the sole use of the protein G column.

The peptide cartography obtained with the MALDI was incomplete probably because of a lack of ionization efficiency. The electrospray ionization analysis led to the detection of multicharged peptides and seemed therefore adapted to this problematic. The highly resolutive Orbitrap analyzer allowed an accurate detection of the proteotypic peptides. This detection was satisfying in the mixture of plasma samples with distinct G3m alleles (Figure 3): the concomitant increases and decreases of two proteotypic peptides contained in preparations which were mixed at sequential ratios allowed validating the relative quantification in a label free approach. In spite of some improvements that are yet to be brought, this study represents the first demonstration of the technical feasibility of detecting peptides representing distinct G3m and IGHG3 alleles.

The detection of peptide WQ**Q**GN**I**FSCSVMHEALHNR (#24, G3m5*) in the samples of the mother-child pair (BEM1 and BEI1) presents the same profile in the Orbitrap and the triple quadrupole in SRM mode (Figure 4). Peptide WQ**E**GN**V**FSCSVMHEALHNR (#26, G3m24*) was detected and identified in several samples (e.g., BEC2, BEC3, BEC4, BEM2), but it was not, although expected from serological data, in the mother and child samples (BEM1, BEC1). Two explanations may be proposed to account for the absence of this peptide: 1) BEM1 brought effectively only the G3m5* allele or 2) it could not be detected on account of insufficient quantity. The first hypothesis considers the sensitivity of the standard hemagglutination inhibition method. As a reminder, this method is qualitative. Its advantage is to detect Gm allotype epitopes but at the same time this makes the results dependent on the specificity of the reagent anti-allotype antibodies and on visual interpretation. As regards the second hypothesis, the quantities of the distinct G3m allele peptides contained in a heterozygous individual sample could be impacted by the purification process. To validate this possibility a relative or absolute quantification of peptides could be processed by mass spectrometry on each purification fraction as well as directly on plasma samples. Another explanation could be that an individual heterozygous for G3m alleles could express differently each of them, as shown for the G1m alleles [26], despite the fact that Gm allotypes are encoded by codominant genes [8]. This possibility was reinforced by the results of the absolute quantification of the 2 peptides detected in the BEC2 sample, WQ**Q**GN**I**FSCSVMHEALHN**R** (#24, G3m5*) and WQ**E**GN**V**FSCSVMHEALHN**R** (#26, G3m24*), for which the concentrations were 56.0 and 990.3 fmol/µL, respectively, with comparable ionization efficiency of the SRM analysis. In the mother-child samples (BEM1, BEI1), WQ**E**GN**V**FSCSVMHEALHN**R** (#26, G3m24*) may have been present but in quantities below the signal/background threshold. The triple quadrupole in SRM mode would be the adequate equipment in the cases where the measurement of an absolute quantity of peptides would be necessary.

The G3m and IGHG3 allele identification based on mass spectrometry may be applied at distinguishing maternal from neonatal antibodies in plasma samples from infants. In their first months of life, newborns are protected by maternal antibodies transferred through the placenta. After a few months, the concentration of maternal antibodies decreases in the infant’s plasma, giving way to neo-synthesized neonatal antibodies.

Quantification of polymorphic peptides for the detection of G3m and IGHG3 alleles may allow following the IgG3 expression in neonates provided that the IGHG3 polymorphisms from the mother and her child are distinct. Restrictions encountered in the case of identical IGHG3 alleles in both mother and child can be overcome by the recourse to the IGHG1 polymorphism alleles and, although less informative, to the IGHG2 and IGHG4 alleles [8], thus reinforcing the capacity to distinguish maternal from infant IGHG alleles. In a recent publication, Goetze and co-workers [26], using similar MS tools, have circled peptides of close homology to those investigated here, from IGHG1 and IGHG2, however this work has mostly focused on glycosylation but not on quantification. The detection and quantification of the neonate’s own antibody response might be essential in some clinical cases in terms of indirect diagnosis. In the case of infections of the newborns [27]–[30], it may be foreseen that the specific IgG produced in neonates be followed, providing by their decrease, information on the response to therapeutic treatment.
